# Enduring Lagrangian coherence of a Loop Current ring assessed using independent observations

**DOI:** 10.1038/s41598-018-29582-5

**Published:** 2018-07-26

**Authors:** Francisco J. Beron-Vera, María J. Olascoaga, Yan Wang, Joaquín Triñanes, Paula Pérez-Brunius

**Affiliations:** 1 Department of Atmospheric Sciences, Rosenstiel School of Marine and Atmospheric Science, University of Miami, Miami, Florida USA; 2 Department of Ocean Sciences, Rosenstiel School of Marine and Atmospheric Science, University of Miami, Miami, Florida USA; 3 Department of Atmospheric and Ocean Sciences, University of California Los Angeles, Los Angeles, California USA; 4 Atlantic Oceanic and Atmospheric Laboratory, National Oceanic and Atmospheric Administration, Miami, Florida USA; 5 Centro de Investigación Científica y de Educación Superior de Ensenada, Ensenada, Baja California Mexico; 6 Cooperative Institute for Marine and Atmospheric Studies, University of Miami, Miami, Florida USA; 7 Instituto de Investigaciones Tecnológicas, Universidad de Santiago de Compostela, Santiago, Spain

## Abstract

Ocean flows are routinely inferred from low-resolution satellite altimetry measurements of sea surface height assuming a geostrophic balance. Recent nonlinear dynamical systems techniques have revealed that surface currents derived from altimetry can support mesoscale eddies with material boundaries that do not filament for many months, thereby representing effective transport mechanisms. However, the long-range Lagrangian coherence assessed for mesoscale eddy boundaries detected from altimetry is constrained by the impossibility of current altimeters to resolve ageostrophic submesoscale motions. These may act to prevent Lagrangian coherence from manifesting in the rigorous form described by the nonlinear dynamical systems theories. Here we use a combination of satellite ocean color and surface drifter trajectory data, rarely available simultaneously over an extended period of time, to provide observational evidence for the enduring Lagrangian coherence of a Loop Current ring detected from altimetry. We also seek indications of this behavior in the flow produced by a data-assimilative system which demonstrated ability to reproduce observed relative dispersion statistics down into the marginally submesoscale range. However, the simulated flow, total surface and subsurface or subsampled emulating altimetry, is not found to support the long-lasting Lagrangian coherence that characterizes the observed ring. This highlights the importance of the Lagrangian metrics produced by the nonlinear dynamical systems tools employed here in assessing model performance.

## Introduction

The prevalent looping behavior of satellite-tracked surface drifting buoys and abundance of persistently closed streamlines of the satellite altimetry sea surface height (SSH) field suggest that eddies with water trapping capability are ubiquitous in the ocean^1,2^. However, owing to the observer-dependent nature of position and velocity, this is only a loose and generally incorrect assessment of Lagrangian (i.e., material) coherence^3–6^.

To assess Lagrangian coherence irrespective of the observer, a specialized technique is necessary. Recent developments in the area of nonlinear dynamical systems have led to such a technique, enabling objective detection from a finite-time-aperiodic flow realization of eddies with material boundaries that experience no filamentation over the detection interval^4,7–10^. Detecting eddies with that property is important because of the impact they may have in global ocean transport^11–14^.

Long-lived *coherent Lagrangian eddies* have been extracted from flows derived geostrophically from satellite altimetric measurements of SSH in a number of occasions^12,13,15,16^. While satellite altimetry is widely used to monitor global ocean variability^17–19^, the long-range Lagrangian coherence assessed for mesoscale eddies detected from altimetry is constrained by the impossibility of current altimeters to resolve submesoscale processes, which are increasingly believed to be important in the upper ocean^20–22^. While the mesoscale dynamics still play a dominant stirring role^23–27^, submesoscale processes may contribute to prevent Lagrangian coherence from manifesting in the rigorous form predicted by the nonlinear dynamical systems theories.

The goal of this paper is to provide support for the enduring Lagrangian coherence of a mesoscale eddy using an unprecedented combination of observations from independent sources. We first detect from altimetry an exceptionally persistent coherent Lagrangian Loop Current ring (LCR) in the Gulf of Mexico (GoM). Then we show that associated with the ring is a satellite-derived chlorophyll deficient patch, confirming the material nature of the ring. Using satellite-tracked surface drifter trajectories we provide evidence that the boundary of the ring is largely convex (wiggle free), showing no significant signs of the presence of submesoscale eddies rolling up around its material boundary.

We also analyze the output from the operational U.S. Navy Coastal Ocean Model (NCOM) of the GoM. At 1-km-horizontal resolution, this data-assimilative system marginally resolves submesoscales. We show that neither the velocity field derived by subsampling the model’s SSH simulating satellite altimetry nor the total upper-ocean model velocities supports a coherent Lagrangian ring with similar characteristics. The simulated altimetry flow produces a smaller and much shorter lived coherent Lagrangian ring, and the total model flow does not sustain a coherent Lagrangian ring, neither at the surface nor below of it within the first 50 m. The latter, however, do reveal the presence of swirling Lagrangian structures that are coherent but in a relaxed sense recently proposed^28^. [The work of Beron-Vera *et al*.^28^ relies to a large extent on the results from the present paper, which was submitted for publication elsewhere several months before Beron-Vera *et al*.’s^28^ work was submitted. Beron-Vera *et al*.^28^ reference an earlier version of this paper under title the “On the significance of coherent Lagrangian eddies detected from satellite altimetry,” posted by the authors in arXiv:170406186].

## Methods

### Coherent Lagrangian eddies

**Figure 1:**
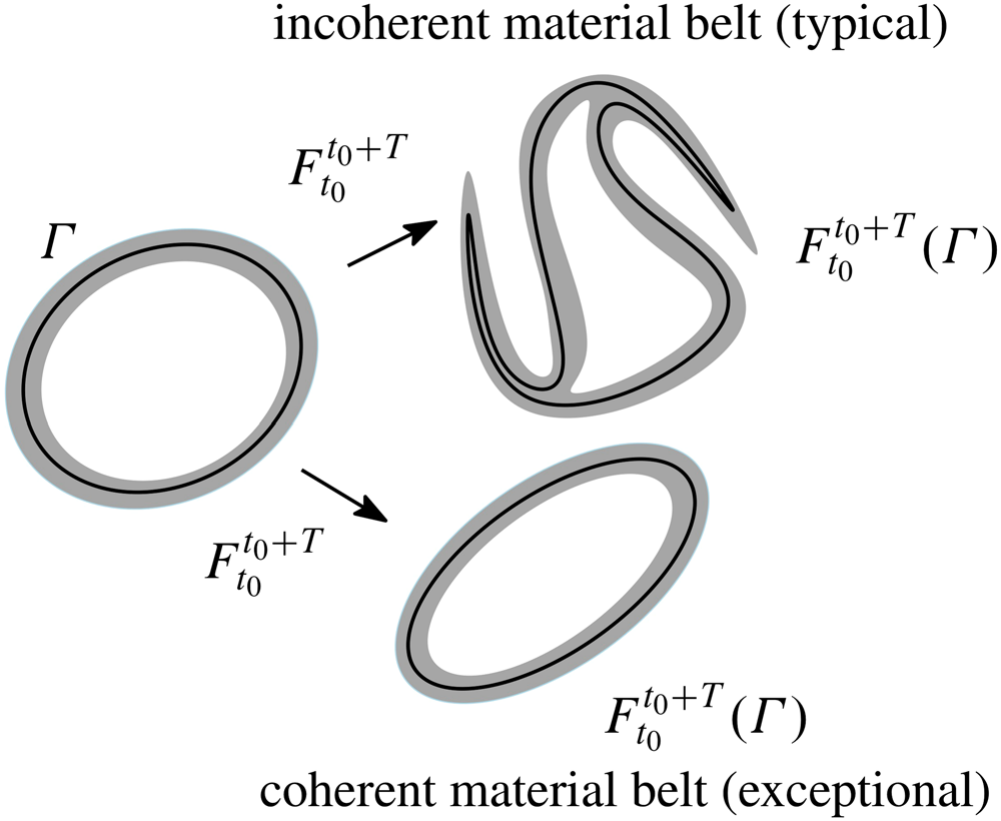
A material loop Γ (black) at time t_0_ is advected by a two-dimensional flow into its later position ${F}_{{t}_{0}}^{{t}_{0}+T}({\rm{\Gamma }})$ at time t_0_ + *T*. The advected material loop remains coherent if an initially thin material belt (grey) around the material loop experiences no leading-order variations in averaged material stretching upon advection. The outermost member in a family of nonintersecting such material loops constitutes the optimal boundary of a coherent material eddy. Figure constructed using Ipe (http://ipe.otfried.org/).

Haller and Beron-Vera^8,9^ seek fluid regions enclosed by exceptional material loops that defy the exponential stretching that a typical loop will experience in turbulent flow. As demonstrated by these authors, such loops, which constitute a type (elliptic) of Lagrangian coherent structures^29^, have small annular neighborhoods exhibiting no leading-order variation in averaged material stretching (Fig. 1).

Solving the above variational problem reveals that the loops in question are uniformly stretching: any of their subsets are stretched by the same factor *λ* under advection by the flow from time *t*_0_ to time *t*. The time *t*_0_ positions of *λ*-stretching material loops turn out to be limit cycles of one of the following two equations for parametric curves $s\mapsto {x}_{0}(s)$:

1 $$\frac{{\rm{d}}{x}_{0}}{{\rm{d}}s}=\sqrt{\frac{{\lambda }_{2}({x}_{0})-{\lambda }^{2}}{{\lambda }_{2}({x}_{0})-{\lambda }_{1}({x}_{0})}}\,{\xi }_{1}({x}_{0})\pm \sqrt{\frac{{\lambda }^{2}-{\lambda }_{1}({x}_{0})}{{\lambda }_{2}({x}_{0})-{\lambda }_{1}({x}_{0})}}\,{\xi }_{2}({x}_{0}),$$

where *λ*_1_(*x*_0_) < *λ*^2^ < *λ*_2_(*x*_0_). Here {*λ*_*i*_(*x*_0_)} and {*ξ*_*i*_(*x*_0_)}, satisfying $0 < {\lambda }_{1}({x}_{0})\equiv {\lambda }_{2}{({x}_{0})}^{-1} < 1$, *ξ*_*i*_(*x*_0_) · *ξ*_*j*_(*x*_0_) = *δ*_*ij*_ (*i*, *j* = 1, 2), are eigenvalues and (normalized) eigenvectors, respectively, of the Cauchy–Green tensor, ${C}_{{t}_{0}}^{t}({x}_{0}):={\rm{D}}{F}_{{t}_{0}}^{t}{({x}_{0})}^{{\rm T}}{\rm{D}}{F}_{{t}_{0}}^{t}({x}_{0})$, an objective (i.e., observer-independent) descriptor of material deformation, where ${F}_{{t}_{0}}^{t}:{x}_{0}\mapsto x(t;{x}_{0},{t}_{0})$ is the flow map that takes time-*t*_0_ positions on a horizontal plane to time-*t* positions of fluid particles, which obey

2 $$\frac{{\rm{d}}x}{{\rm{d}}t}=v(x,t),$$

where *v*(*x*, *t*) is a two-dimensional velocity field.

Limit cycles of (1) either grow or shrink under changes in *λ*, forming smooth annular regions of nonintersecting loops. The outermost member of such a band of material loops is observed physically as the boundary of a *coherent Lagrangian eddy*. Limit cycles of (1) tend to exist only for *λ* ≈ 1. Material loops characterized by *λ* = 1 resist the universally observed material stretching in turbulence: they reassume their initial arclength at time *t*. This conservation of arclength, along with enclosed area preservation, can produce extraordinary coherence^4^. Finally, limit cycles of (1) are (null) geodesics of the generalized Green–Lagrange tensor ${C}_{{t}_{0}}^{t}({x}_{0})-{\lambda }^{2}{\rm{I}}d$, which must necessarily contain degenerate points of ${C}_{{t}_{0}}^{t}({x}_{0})$ where its eigenvector field is isotropic. For this reason the above procedure is known as *geodesic eddy detection*.

The numerical implementation of geodesic eddy detection is documented at length^7–9,12,13,15,29–32^. A software tool is also available^33^. Here we have set the grid spacing of the computational domain to 0.1 km^12,13,23,27,28^. All integrations were carried out using a step-adapting fourth/fifth-order Runge–Kutta method with interpolations done with a cubic method.

We close this section by noting that while geodesic eddy detection is two-dimensional in nature, it can be applied (as done below) levelwise two-dimensionally to gain insight into three-dimensional aspects of the flow as described for instance by a hydrostatic model like NCOM.

### Data

The SSH is taken as the sum of a (steady) mean dynamic topography and the (transient) altimetric SSH anomaly. The mean dynamic topography is constructed from satellite altimetry data, *in-situ* measurements, and a geoid model^34^. The SSH anomaly is provided weekly on a 0.25°-resolution longitude–latitude grid. This is referenced to a 20-year (1993–2012) mean, obtained from the combined processing of data collected by altimeters on the constellation of available satellites^35^.

The ocean color data consist of chlorophyll concentration fields obtained from the National Aeronautics and Space Administration (NASA) Biology Processing Group website (http://oceancolor.gsfc.nasa.gov). Sun glint and clouds represent a major source of data gaps in the GoM, especially during the summer months. To minimize their effects and improve coverage, we have chosen to use multiday Level-3 mapped products. These are 8-day composite chlorophyll fields at 4-km horizontal resolution from the MODIS (Moderate Resolution Imaging Spectroradiometer) sensor, on-board NASA’s Earth Observing System (EOS) *Terra* and *Aqua* satellites, and the VIIRS (Visible Infrared Imaging Radiometer Suite) sensor, aboard the *Suomi-NPP* satellite.

The surface drifters are Far Horizon Drifters (FHD), manufactured by Horizon Marine, Inc.^36,37^. These are deployed by Horizon Marine Inc. as part of the EddyWatch program. A subset of 7 to 16 drifters deployed within or in the closest vicinity of the LCR under investigation are considered. Each drifter consists of a cylindrical buoy attached to a 45-m tether line, attached itself to a 1.2-m “para-drogue.” These instruments are deployed by air, so the drogue serves both as parachute to protect the buoy when air deployed, and to reduce wind slippage of the buoy as it drifts in the water. Positions are recorded hourly using GPS that transmit the data via the *Argos* system. The quality control of the data consisted of interpolating positions between data gaps shorter than 6 hours, and removing locations over land and data points with speeds exceeding 3 m s^−1^. Correlations of the drifter velocities with winds^38^ are of the same order as those obtained with the SVP drifters^39^, which are drogued with holey socks at about 15 m. This suggests that wind-slippage on the FHD drifter is minimal and the water-following characteristics of FHD drifters are in general similar to the drifters used by the National Oceanic and Atmospheric Administration (NOAA) Global Drifter Program^40^.

### Model

The NCOM simulation employs assimilation and nowcast analyses from NCODA (Navy Coupled Ocean Data Assimilation)^41^. Forecasts are generated by systems linking NCODA with regional implementations^42^ of NCOM^43^. The model has 1-km horizontal resolution and was initiated on 15 May 2012 from the then operational global ocean model Global Ocean Forecast System (GOFS) 2.6^44^. Daily boundary conditions are received from the current operational GOFS using the HYbrid Coordinate Ocean Model (HYCOM)^45^. The vertical grid is comprised of 49 total levels; 34 terrain-following *σ*-levels above 550 m and 15 lower *z*-levels. The *σ*-coordinate structure has higher resolution near the surface with the surface layer having 0.5-m thickness. The simulation uses atmospheric forcing at the sea surface from COAMPS (Coupled Ocean/Atmosphere Mesoscale Prediction System)^46^ to generate forecasts of ocean state up to 72 h in 3-hour increments. The observational data assimilated in these studies is provided by NAVOCEANO (Naval Oceanographic Office) and introduced into NCODA via its ocean data quality control process. Observations are three-dimensional variational (3DVar) assimilated^47^ in a 24-hour update cycle with the first guess from the prior day NCOM forecast.

## Results

The focus of our analysis is a region filled with closed streamlines of the altimetric SSH field in the GoM, which can be tracked for nearly one year since April 2013. The region was classified as an LCR by the Horizon Marine Inc.‘s EddyWatch program (http://www.horizonmarine.com) using Leben’s^48^ methodology, which searches for Eulerian footprints of LCRs on the SSH field, and further named *Kraken*.

**Figure 2:**
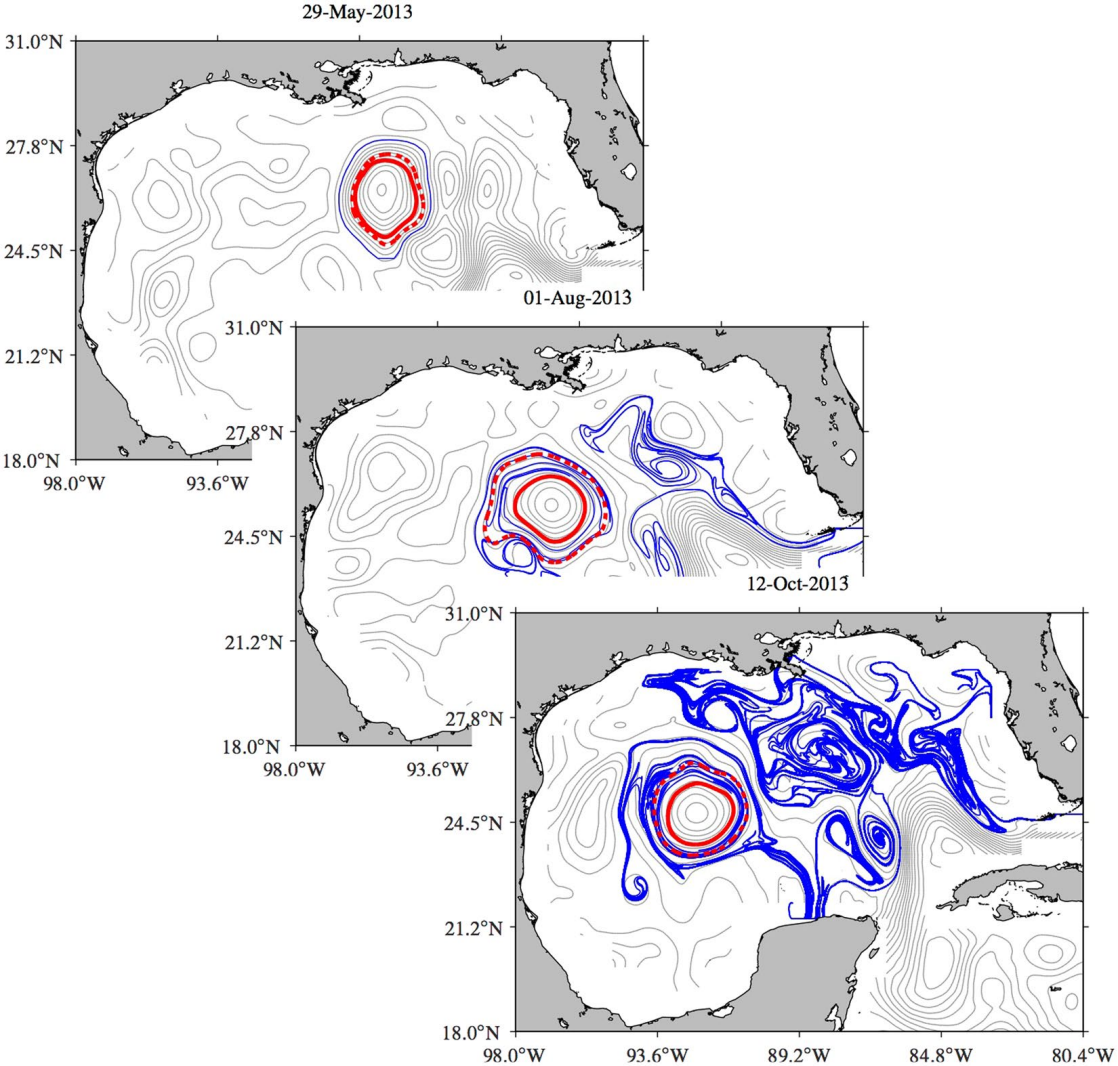
In grey, contour levels of the altimetric sea surface height (SSH) field in the Gulf of Mexico (GoM) on selected days. Overlaid in blue are snapshots of the passive evolution, according to the altimetry-derived flow, of tracers initially on 29 May 2013 along the outermost of the closed SHH streamlines filling a mesoscale region of nearly 100-km radius in the center of the GoM, which has been identified as a Loop Current ring (LCR) and named *Kraken*. These closed instantaneous SSH streamlines are the Eulerian footprints of a coherent Lagrangian LCR, which nearly steadily translates westward across the GoM. The solid red curve is the boundary for the longest-lived and largest coherent Lagrangian LCR core. The dashed red curve is a material loop that provides repeated, short-term shielding to this core. Figures constructed using MATLAB R2017b (http://www.mathworks.com/).

Figure 2 shows in grey several contour levels of the SSH field on three selected days, progressing from top to bottom. Highlighted in blue on the top is the outermost closed SSH contour in the region identified as LCR *Kraken* on the indicated day. Two images of this curve under the flow map associated with the altimetry-derived advection field are shown on the middle and bottom. More specifically, these are the result of integrating the passive tracer particle equation (2), with the velocity derived geostrophically from SSH, from tracer positions initially on 29 May 2013 along the curve. Several additional frames, including instantaneous SSH isolines as well as advected images of the tracer curve over several months, are shown in Supplementary Movie S1.

Note the filamentation experienced by the passively advected tracer curve. Note also that, while intense, the filamentation is mainly outward. The outward-filamented curve portion reveals a number of swirling flow regions of varied sizes in the mesoscale range resolved by altimetry and also below, as a result of nonlocal straining by the altimetry-derived flow. Most of these swirls are not seen to preserve their entity over time. The inward-filamented curve piece, by contrast, reveals a enduring compact region, of a mean radius of nearly 100 km, contained inside the region where the SSH streamlines are instantaneously closed and has been identified as LCR *Kraken*. Two key observations about the behavior of the tracer near this region are in order. First, the tracer does not invade the region over the course of many months. Rather, it wraps around the region in an anticyclonic fashion. Second, the wrapping tracer tends to occupy a thick belt (with a width of 10 to 20 km) around the region in question. These two observations suggest on one hand that LCR *Kraken* has an impermeable material core, surrounded by a resilient material loop which acts as a long-term barrier for the transport of tracer into and out from the material core. On the other hand, outside this long-term closed transport barrier, shorter-term material barriers must be emerging repeatedly, shaping and confining the tracer wrapping around the long-term coherent Lagrangian ring core.

Geodesic eddy detection has been designed to unveil the precise location of such long- and short-term resilient material loops. These are indicated by a solid and dashed red curve, respectively, in Fig. 2. To unveil them, we applied geodesic eddy detection on the altimetry-derived velocity field over the time interval [*t*_0_, *t*_0_ + *T*] for appropriate choices of *t*_0_ and *T*.

The specific choices *t*_0_ = 29 May 2013 and *T* = 200 d were found to produce a resilient material loop that provides the boundary for the largest and longest-lived coherent Lagrangian ring core. From *t*_0_ = 29 May 2013 to *t*_0_ + *T* = 15 December 2013, this boundary uniformly stretches by a factor *λ* = 1.05. This minor uniform stretching of the boundary along with the conservation of the enclosed area, which holds because the velocity field is geostrophic and hence divergenceless, conveys LCR *Kraken* exceptional Lagrangian coherence.

Shorter-term resilient material loops encompassing the largest area which includes the long-term coherent Lagrangian ring core are found from the application of geodesic eddy detection every *t*_0_ from 29 May daily through 15 December 2013 using *T* = 30 d. These emerging material loops reassume their initial arclength (i.e., they have *λ* = 1) after a period of one month. As such, they convey additional temporary but repeated shielding to the ring core, reinforcing its coherent Lagrangian nature. Successive short-term coherence regain events where first reported by Wang *et al*.^13^ for Agulhas rings in the South Atlantic.

Beyond the fact that our inference on the exceptional Lagrangian coherence of LCR *Kraken* remains valid irrespective of the reference frame adopted by the observer, a unique aspect of geodesic eddy detection, the pertinent question that arises is whether this is a real ingredient of the ocean circulation in the GoM or is just an artifact of the limitation of satellite altimetry to resolve submesoscale and likely ageostrophic motions. It turns out that, as we proceed to show, LCR *Kraken* constitutes to a large extent a realistic coherent Lagrangian ring. We support our assertion with two pieces of independent observations, rarely available simultaneously over an extended period of time.

**Figure 3:**
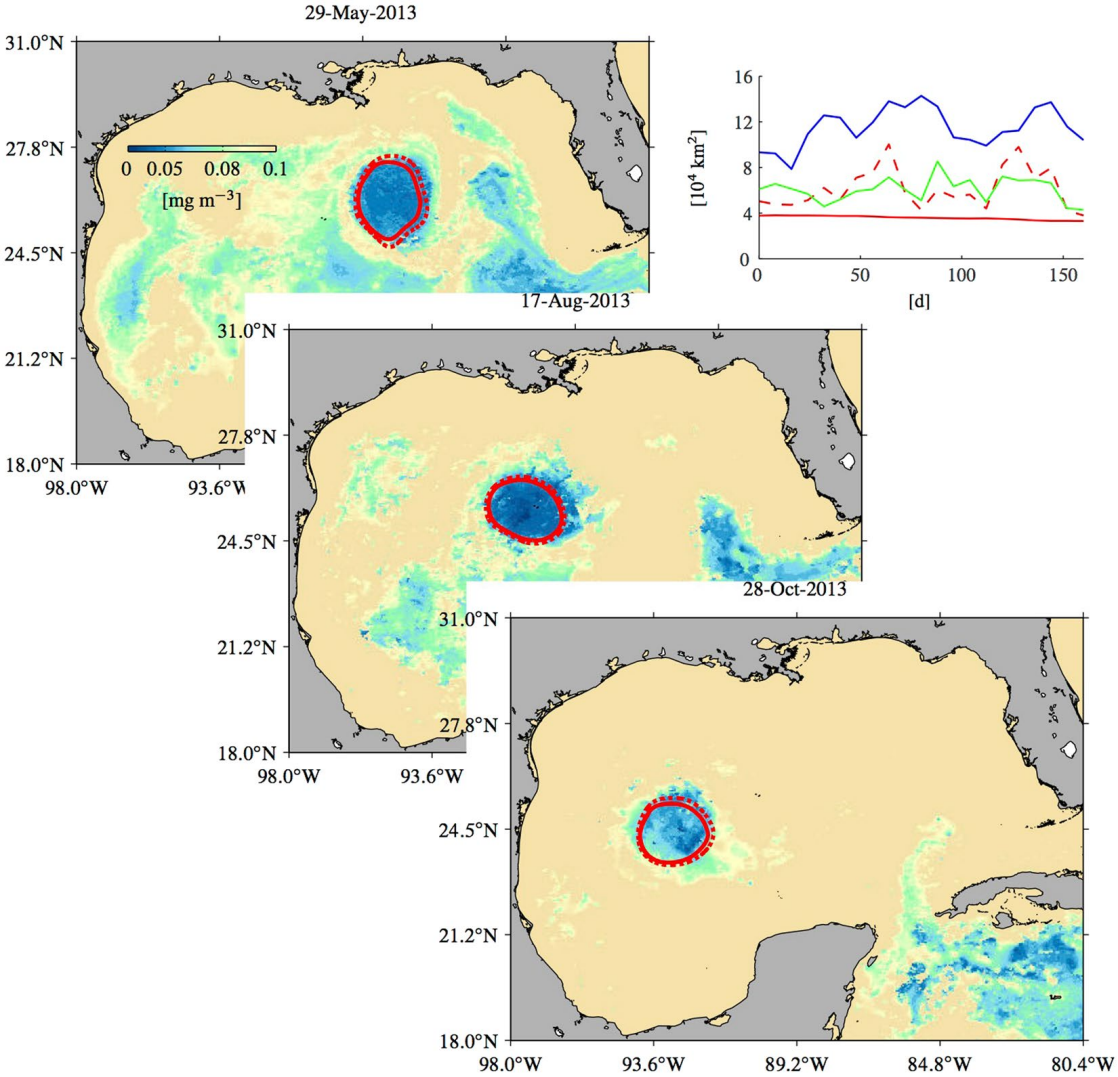
Satellite-derived chlorophyll concentration images on selected days with the altimetry-inferred long- (solid) and short-term (dashed) coherent Lagrangian boundaries of LCR *Kraken* overlaid. The top-right panel shows, as a function of time since 29 May 2013, area enclosed by the long- (solid red) and short-term (dashed red) Lagrangian boundaries, the outermost instantaneous closed SSH contour level (blue), and the area spanned by the chlorophyll deficient patch (green). Figures constructed using MATLAB R2017b (http://www.mathworks.com/).

The first piece of observations is given by satellite ocean color. Figure 3 shows snapshots of chlorophyll concentration on selected days with the altimetry-inferred long- and short-term coherent Lagrangian ring boundaries overlaid (solid and dashed red, respectively). Weekly snapshots in the period 19 April through 21 November 2013 are shown in Supplementary Movie S2.

Note the patch of low chlorophyll concentration which, for a period as long as about seven months, quite closely accompanies the altimetry-inferred coherent Lagrangian ring as it nearly steadily translates westward in the GoM. Note also that the origin of the chlorophyll defect can be traced completely into the Caribbean Sea, which is characterized by predominantly oligotrophic conditions^49^. This indicates that the formation of LCR *Kraken* follows the standard scenario: the Loop Current occludes and LCR *Kraken* is subsequently pinched off from the current. This process is Lagrangian in essence^50^. Altogether the two observations just made provide strong support to our conclusion that LCR *Kraken* is largely Lagrangian.

Clearly, some deviations between the boundaries of the low-chlorophyll patch and the altimetry-inferred coherent Lagrangian ring are evident. But these are expected because of at least two reasons. First, cloud coverage is important at times during the observational period. This results in nonuniform sampling, which impacts the interpolation of the data on a regular grid and hence the resolution of the chlorophyll defect. Second, chlorophyll as a tracer cannot be considered to be entirely passive. Vertical pumping of nutrients, not represented by the altimetry-derived flow, at the periphery of LCR *Kraken* may be expected^51^. Biological activity stimulated by nutrient availability may be modifying the chlorophyll concentration and thus the size of the patch as it translates across the GoM.

However, the geodesically detected long- and short-term Lagrangian boundaries much better constrain the extent of the chlorophyll deficient patch than instantaneous closed streamlines of the SSH field. This is demonstrated in the top-right panel of Fig. 3, which compares, as a function of time since 29 May 2013, the area enclosed by the long- (solid red) and short-term (dashed red) Lagrangian boundaries and the outermost instantaneous closed SSH contour level (blue) with the area spanned by the chlorophyll deficient patch (green), whose boundary was determined as the chlorophyll isoline where the concentration change with respect to the area enclosed by it is highest. Note that the area of the chlorophyll deficient patch is not constant as is (to numerical error) that enclosed by the long-term boundary. While biological activity is likely acting to shape the form and extent of the patch, this action is constrained by the Lagrangian circulation, which favors coherence regain events that prevent the chlorophyll depleted water from spreading away from LCR *Kraken*.

**Figure 4:**
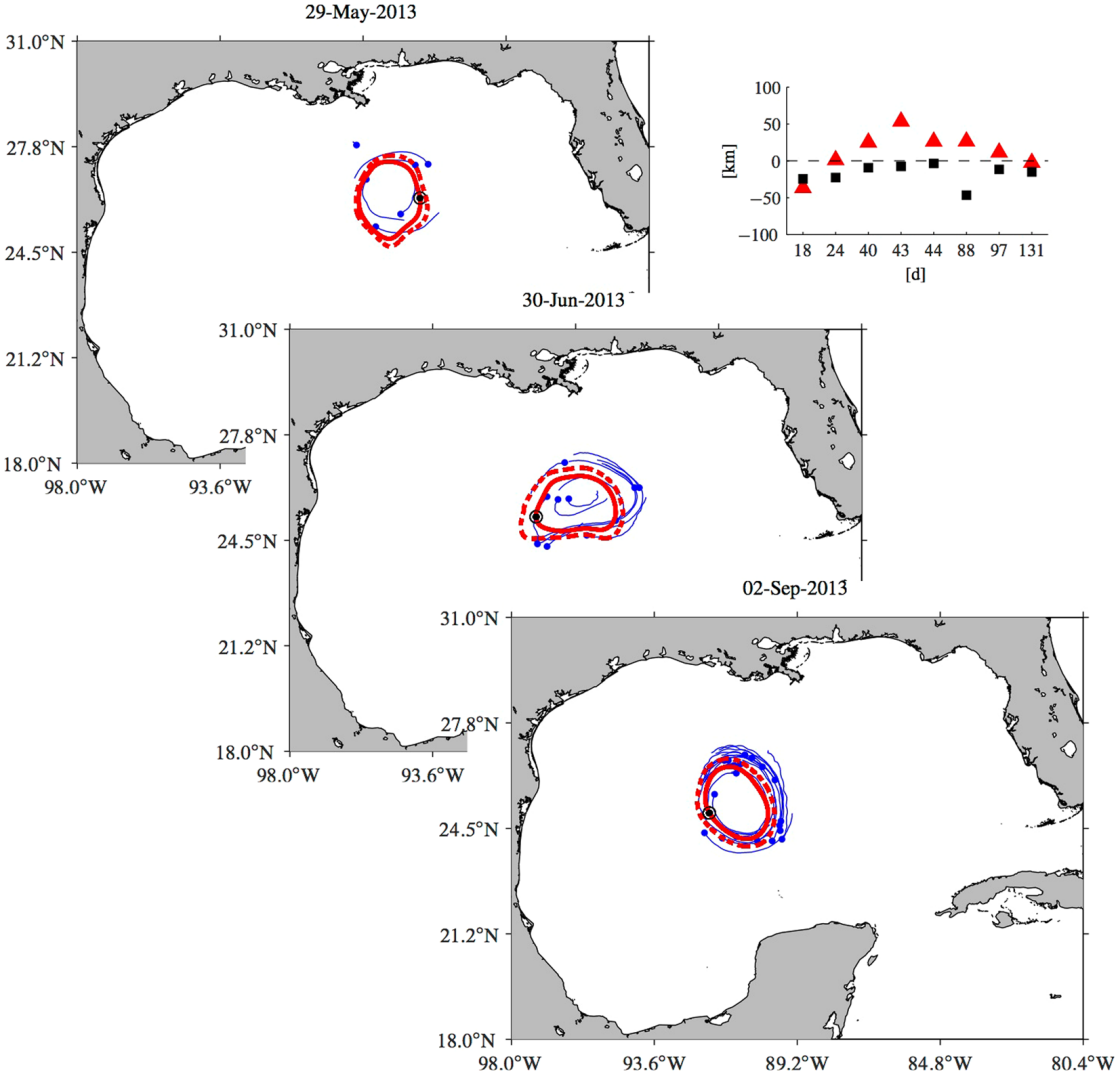
Snapshots of the evolution of the altimetry-inferred coherent Lagrangian long- (solid red) and short-term (dashed red) boundaries of LCR *Kraken* along with that of satellite-tracked surface drifters initially as close as 50 km from the long-term boundary (blue). The drifter positions on each day shown are indicated by blue dots. The tails attached to the dots are week-long past trajectory segments. One point on the long-term boundary is highlighted by a black dot surrounded by a circle, for reference. The top-right panel shows as a function of drifter lifespan, initial (black square) and mean (red triangle) shortest signed distance to the long-term boundary for all drifters initially lying within. Figures constructed using MATLAB R2017b (http://www.mathworks.com/).

The second piece of observations is provided by trajectories of satellite-tracked surface drifters. As part of the routine monitoring of LCRs, the Horizon Marine Inc.’s EddyWatch program deployed various drifters in the vicinity of LCR *Kraken* along its westward path. Figure 4 shows snapshots of the evolution of the altimetry-inferred coherent Lagrangian ring long- and short-term boundaries, indicated by solid and dashed red curves, respectively, along with that of drifters initially lying within these boundaries or which at some point in time have come as close as 50 km to the long-term boundary (various additional snapshots are shown in Supplementary Movie S3). One point on the long-term boundary is highlighted by a black dot surrounded by a circle, for reference. The drifter positions are indicated by blue dots on the specified day. The tails (in blue) attached to the dots are week-long past trajectory segments.

The first aspect to note is that the drifters deployed inside or near by the long- and short-term boundaries tend to remain in their vicinity, very closely following the anticyclonic circulatory motion of passive tracers along them. This can be most easily visualized by comparing the position of the point highlighted on the long-term boundary and those of the nearby drifters. The turnover timescale of that point is of about two weeks, which is nearly the same for the drifters in its vicinity. Overall the behavior of the drifters is largely consistent with the motion of tracer particles passively advected by the altimetry-derived flow. This provides further support to the Lagrangian nature of LCR *Kraken* inferred from the analysis of altimetry data.

A quantitative assessment of the above qualitative observations is provided in the top-right panel of Fig. 4, which shows, as a function of drifter lifespan, initial (black square) and mean (red triangle) shortest signed distance to the long-term coherent Lagrangian boundary of LCR *Kraken* for all drifters initially lying within (a negative value indicates that the drifter is inside the boundary). Two out of 8 drifters spent their whole lifespan (18 and 131 d) within the long-term boundary. The other 6 drifters remained around the ring with a mean minimum distance shorter than 50 km.

Inertial effects (i.e., of objects’ finite size and buoyancy) might explain the behavior of the relatively slow outward moving drifters. Indeed, floating objects are predicted to be repelled away from anticyclonic coherent Lagrangian eddies^10,15^. These effects can be expected to be most effective when the para-drogues attached to the drifters are closed. The outward motion of the drifters may be indicative of this. Windage may also explain deviations of drifter motion from purely Lagrangian motion. However, windage effects near mesoscale eddies or rings have not been well constrained for biological tracers^51–53^, and they may be outbalanced by inertial effects^54^.

An additional important aspect to be highlighted of the behavior of the drifters is the relative absence of submesoscale wiggles in their hourly-sampled trajectories. The lack of such wiggles in the drifter trajectories is largely consistent with the convexity of the altimetry-inferred long- and short-term coherent Lagrangian ring boundaries. This observation gives further support to the significance of the altimetry-based inference.

It might be argued that the dominance of wiggle-free drifter trajectories is a consequence of the drifters being para-drogued at about 50-m depth, which lies somewhat below the summertime mixed-layer base^55^. However, this should not prevent the drifters from sampling submesoscale motions if they were active^2,38^. Indeed, loops of about 10-km or smaller diameter can be seen at times mounted on the drifter trajectories. But the frequency of those loops is typically very close to the local Coriolis frequency, as it has been observed earlier in the same region using the same drifters^37^. So the loops mainly correspond to near-inertial oscillations, which do not impact relative dispersion statistics^24^ and thus should not be expected to constitute an effective mechanism for the erosion of mesoscale coherent Lagrangian structures such as the resilient material boundary around LCR *Kraken*.

**Figure 5:**
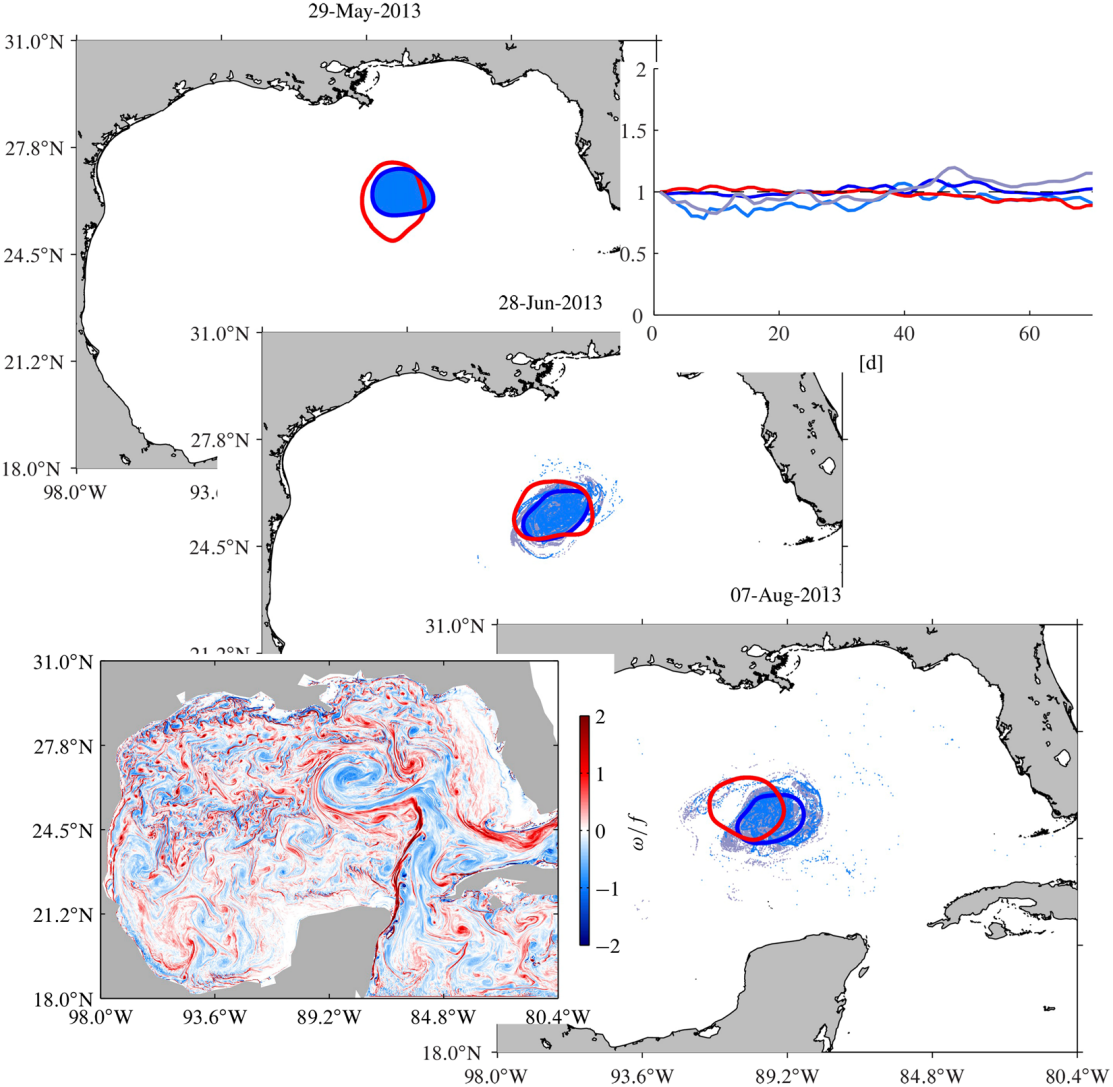
Snapshots of the evolution of the boundary of coherent Larangian LCR *Kraken* as extracted from altimetric SSH (red) and subsampled SSH from a submesoscale permitting Navy Coastal Model (NCOM) simulation of the GoM (blue), and a tracer patch passively advected by the total model velocity at the surface (light blue) and at 50-m depth (gray). The top-right panel shows the mean distance to the corresponding centroid from points on the altimetry-derived boundary (red), the model-SSH-derived boundary (blue), and the boundary of the tracer patch passively advected by the total model velocity at the surface (light blue) and at 50-m depth (gray), each normalized by the value taken initially on 29 May 2013. The bottom-left panel is snapshot of the surface vorticity (normalized by the mean Coriolis parameter) field on 29 May 2013. Figures constructed using MATLAB R2017b (http://www.mathworks.com/).

It is still of interest to investigate if mesoscale coherent Lagrangian eddies can be realized in the presence of submesoscale motions. We carried such an investigation by seeking evidence of coherent Lagrangian LCR *Kraken* in the data-assimilative submesoscale-permitting NCOM simulation of the GoM. The bottom-left panel of Fig. 5 shows a snapshot of the surface vorticity (*ω*) normalized by the mean Coriolis parameter in the GoM (*f*) from this simulation, which reveals a large number of eddy-like features spanning scales in the meso- and submesoscale ranges. Note in particular the suprainertial (|*ω*|/*f* > 1) submesoscale eddies rolling around the mesoscale eddy feature in the center of the GoM, which appears to be the signature of LCR *Kraken* in the model vorticity field.

However, we have not been able to extract a mesoscale coherent Lagrangian ring akin to LCR *Kraken* from the NCOM upper ocean (surface, 10, and 50-m levels) velocity field on any day and for any coherence timescale choice or stretching parameter. But we have found that a velocity field computed geostrophically using subsampled model SSH does sustain one. The procedure employed to obtain the subsampled SSH field emulates that used to construct an SSH field by interpolating along-track satellite altimetry measurements^56^. Figure 5 shows snapshots of the evolution of the extracted coherent Lagrangian boundary (dark blue) along with the boundary obtained from satellite altimetry (red). The area enclosed by the simulated ring boundary is 1.6 times smaller than that enclosed by the observed boundary. The centroid of the simulated ring relative to that of the observed ring varies from no less that 10 km to as much as 85 km over 80 d since the extraction date. Over that period the observed ring translates mainly westward, while the simulated ring translates mainly southwestward. The mean westward translation speed for the observed ring is about 3.5 km d^−1^, while that of the simulated ring is nearly 2 km d^−1^. Note that shallow-water lenses on a *β*-plane translate westward at a seeped of ref.^57^ $\beta {L}_{{\rm{D}}}^{2}\approx 3.6$ km d^−1^ for a typical Rossby deformation scale in the GoM of 45 km^58^. This agrees well with the westward translation speed of the ring extracted from satellite altimetry. However, the ring extracted using simulated altimetry translates westward more slowly, at nearly half the predicted speed. Furthermore, the coherence horizon for the simulated ring is *T* = 80 d, which implies a much weaker long-range transport ability for this ring than for the observed ring, whose coherence timescale is *T* = 200 d.

**Figure 6:**
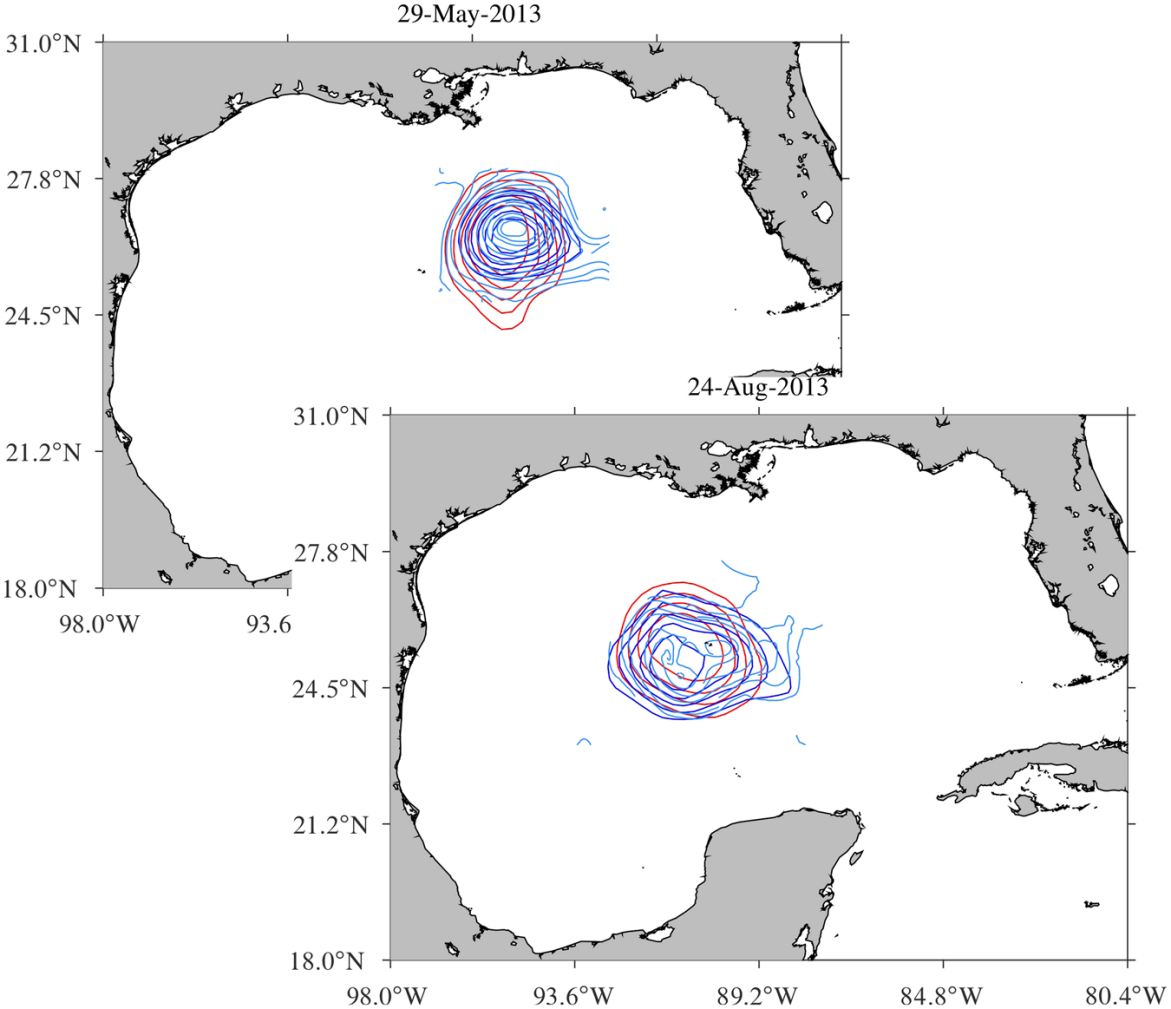
Snapshots of altimetric-SSH-derived (red), model-SSH-derived (blue), and surface model velocity (light blue) streamlines. Figures constructed using MATLAB R2017b (http://www.mathworks.com/).

The above differences in Lagrangian behavior are not obvious from naked-eye comparison of satellite and simulated altimetry SSH or total model surface velocity streamlines. Figure 6 shows snapshots of the latter in red, blue, and light blue, respectively, on nearly three-month apart dates, restricted to the vicinity of the region occupied by the observed LCR. Satellite and model SSH streamlines are closed, nearly concentric at all times. In turn, total model surface velocity also show large (mesoscale) nonintersecting streamline loops, which, unlike altimetry and model SSH streamlines, can encircle smaller (submesoscale) closed as well as open streamline loops. A cursory Eulerian evaluation would indicate mesoscale Lagrangian coherence in all cases simply because the instantaneous streamlines remain closed as time progresses. A more elaborated Eulerian analyisis^59^ would assess the ability of these compact streamline flow regions to self advect by measuring their degree of nonlinearity to rule out the possibility that they represent purely linear wave superpositions. A basic estimate of the degree of nonlinearity of a wave is given by the ratio *U*/*c* of the maximal mean speed *U* at the periphery of a closed streamline region to its translation speed *c*. When *U*/*c* > 1, fluid may be expected to be carried along by a wave, and the wave will in general be dynamically nonlinear, in that its self-advection of momentum or vorticity affects its own evolution. In all cases *U*/*c* > 1, inferring Lagrangian coherence over regions of similar size. But it is possible^3^ to construct examples of flows with closed streamlines at all times for which *U*/*c* > 1 in a given reference frame, while no coherent Lagrangian eddy is present by observing the motion on a frame in which the flow is steady. Thus, given the observer-dependent nature of this Eulerian analysis, it is not surprising that it leads to a coherence assessment at odds with that drawn from the observer-independent Lagrangian analysis carried out here. The nonlinear dynamical systems analysis provides unambiguous means for assessing the performance of a model through various parameters that measure its skill in representing observed Lagrangian behavior. These metrics include the various measures discussed above, namely, coherence timescale, area enclosed, trajectory, and translation speed of Lagrangian rings.

The differences in Lagrangian behavior found are puzzling given that the NCOM simulation was shown to reproduce relative dispersion statistics down into the marginally submesoscale range^24^ as observed during the Grand LAgrangian Experiment (GLAD)^23^. Irrespective of the ability of the model to reproduce observed behavior, a valid question still is whether the simulated total velocity field supports some form of mesoscale Lagrangian coherence. Recall that no coherent Lagrangian ring was possible to be extracted from the total model flow, neither at the surface nor at 10 m or 50 m. The latter lies below the model’s mixed layer during the summer period analyzed, which makes one wonder why velocities at that depth would not support a coherent Lagrangian eddy boundary. However, the mean kinetic energy (about 0.065 m^2^s^−2^) is roughly uniform within the upper 50 m, indicating similarly intense submesoscale activity at the surface and below, inside this layer. With this in mind, we advected under the total model velocity, both at the surface and 50 m, passive tracers initially within the coherent Lagrangian boundary detected from simulated altimetry. This numerical experiment revealed the existence of a swirling Lagrangian structure at the surface (light blue dots in the diagonal panels of Fig. 5), which, while experiencing filamentation, prevailed in large part confined within the interior of the boundary obtained from simulated altimetry. The total model flow at 50 m revealed a similar form of coherence, as it can be seen in the same figure (gray dots). A compactness measure of these structures is provided in the top-right panel of Fig. 5. The compactness measure considered is given by the mean distance from the boundary of a shape with respect to its centroid^60^. The figure shows this distance from points initially on 29 May 2013 lying along the boundary extracted from satellite altimetry and as they are advected by the flow derived from it (red), the boundary obtained from simulated altimetry and as they are advected by the corresponding velocity (blue), and the boundary of the tracer patch and as they are advected by the total model velocity at the surface (light blue) and at 50-m depth (gray), each normalized by the value taken on 29 May 2013. Note that the compactness measure remains close to one for the boundaries extracted from satellite and simulated altimetry, consistent with their coherent Lagrangian nature. The departure from one can be larger for the tracer clouds advected by the total model velocity at the surface and 50 m, but this is never in excess of 0.25.

Important steps toward rigorously characterizing the reported form of mesoscale Lagrangian coherence amid  submesoscale motions have been made in recent work by Beron-Vera *et al*.^28^. Lagrangian coherence in that work is assessed based on the ability of the elements of a material loop to rotate in consonance^10^ or a region to maintain short distances among themselves relative to their distances to particles outside of the region^61^. Clearly, the relevance of such a form of Lagrangian coherence depends on the extent that the intensity of the submesoscale activity in the model can be supported with observations. The observations analyzed here do not provide support for it in the specific region of the GoM and timespan considered.

## Summary and Discussion

In this paper we have provided independent observational support for the enduring Lagrangian coherence of a Loop Current ring (LCR) in the Gulf of Mexico (GoM), which was detected from its Eulerian footprints in satellite altimetry sea surface height (SSH) data and termed *Kraken*. Coherent Lagrangian eddies have material boundaries that do not filament or experience global break away over the coherence assessment time interval. A recent technique rooted in nonlinear dynamical systems theory, geodesic eddy detection, has been specifically designed to unveil these eddies from a velocity realization in an observer-independent fashion.

Applying geodesic eddy detection on the altimetry-derived velocity field, we inferred that LCR *Kraken* possessed a Lagrangian core of about 100-km radius, surrounded by a resilient boundary that remained so for a period as long as seven months. Lagrangian boundaries with similar but shorter-lived resilience, which enclosed a larger domain including this core, were found to emerge over this period. These boundaries conveyed further Lagrangian coherence to LCR *Kraken*.

The observational support for the altimetry-inferred material coherence was obtained from two, rarely available simultaneously, independent sources: satellite ocean color and satellited-tracked surface drifter trajectories. A low-concentration chlorophyll patch traceable into the Caribbean Sea accompanied LCR *Kraken* in its nearly steady westward translation across the GoM. The surface drifters, in turn, developed relatively wiggle-free anticyclonic loops inside or immediately outside LCR *Kraken*’s Lagrangian core with turnover times (of two weeks or so) very close to those of passive tracer particles circling along the boundary of the core.

Recent eddy retention studies^11,62–65^ suggest that the reported Lagrangian supercoherence for LCR *Kraken* may be extended to rings and eddies in other regions of the world oceans. This clearly requires an investigation that exceeds the breadth of the present contribution.

The output from the operational U.S. Navy Coastal Ocean Model (NCOM) of the GoM was finally analyzed in search for evidence of the observed coherent Lagrangian LCR. Neither the flow derived using subsampled model SSH simulating satellite altimetry nor the total upper-ocean model flow was found to support a coherent Lagrangian ring with the same characteristics as the observed coherent Lagrangian LCR. The simulated altimetry revealed a coherent Lagrangian ring which was smaller and much shorter lived than the observed ring, and the total model flow did not support a coherent Lagrangian ring at all, neither at the surface nor within the first 50 m below of it. However, swirling Lagrangian structures, which can be characterized as coherent but in a relaxed sense^28^, were found to be sustained by the total upper-ocean model flow.

The lack of agreement between the simulation and the observed behavior as evidenced by the low model skill as measured by the several Lagrangian metrics resulting from the application of the nonlinear dynamical systems tools is puzzling. On one hand, an altimetry-assimilative system that marginally resolves submesoscales should be able to capture observed mesoscale motions with a reasonable level of accuracy. On the other hand, the model was recently found^24^ to reproduce observed relative dispersion statistics correctly down into to the marginally submesoscale range.

A possible explanation for the undesired behavior of the NCOM simulation may lie in the way that altimetry data are assimilated into the model, which was found^66^ to produce poor agreement between observed and simulated instantaneous Lagrangian transport patterns. The agreement was improved^66^ by dividing the analysis increment fields by the data time window length to more properly weight the data. However, to the best of our knowledge, this fix has not yet been implemented operatively, and is mainly expected^66^ to lead to stronger current fields, which is unclear how they can impact tracer dispersion over time. Another explanation may be found in the increased resolution of the model toward the submesoscale, which can conspire against its mesoscale forecasting skill as it has been recently shown^67^. Discerning among these explanations and possibly additional causes clearly deserves investigation, but this is beyond the scope of this paper.

## Electronic supplementary material

Supplementary Information

Movie 1

Movie 2

Movie 3
