# Supplementary material for: Enduring Lagrangian coherence of a Loop Current ring assessed using independent observations
Source: Sci Rep. 2018 Jul 26;8:11275. doi: 10.1038/s41598-018-29582-5 (PMC6062630; doi:10.1038/s41598-018-29582-5)
Supplement: Supplementary file 1 — Supplementary Information [file 41598_2018_29582_MOESM1_ESM.pdf]

Supplementary Information for “Enduring Lagrangian coherence of a Loop Current ring assessed using independent observations”

Francisco J. Beron-Vera[1,*], María J. Olascoaga[2], Yan Wang[3], Joaquín Triñanes[4,], and Paula Pérez-Brunius[5]**

[1]Department of Atmospheric Sciences, Rosenstiel School of Marine and Atmospheric Science, University of Miami, Miami, Florida, USA.
[2]Department of Ocean Sciences, Rosenstiel School of Marine and Atmospheric Science, University of Miami, Miami, Florida, USA.
[3]Department of Atmospheric and Ocean Sciences, University of California Los Angeles, Los Angeles, California, USA.
[4]Atlantic Oceanic and Atmospheric Laboratory, National Oceanic and Atmospheric Administration, Miami, Florida, USA.
[5]Centro de Investigación Científica y de Educación Superior de Ensenada, Ensenada, Baja California, México.
[*]Corresponding author. E-mail: fberon@rsmas.miami.edu.
[**]Also at Cooperative Institute for Marine and Atmospheric Studies, University of Miami, Miami, Florida, USA; and Instituto de Investigaciones Tecnológicas, Universidad de Santiago de Compostela, Santiago, España.

Supplementary movie legends

Movie S1. In grey, daily contour levels of the altimetric sea surface height (SSH) field in the Gulf of Mexico (GoM) from 29 May through 14 October 2013. Overlaid in blue are snapshots of the passive evolution, according to the altimetry-derived flow, of tracers initially on 29 May 2013 along the outermost of the closed SHH streamlines filling a mesoscale region of nearly 100-km radius in the center of the GoM, which has been identified as a Loop Current ring (LCR) and named *Kraken*. These closed instantaneous SSH streamlines are the Eulerian footprints of a coherent Lagrangian LCR, which nearly steadily translates westward across the GoM. The solid red curve is the boundary for the longest-lived and largest coherent Lagrangian LCR core. The dashed red curve is a material loop that provides repeated, short-term shielding to this core. Animation constructed using FFmpeg (http://www.ffmpeg.com); individual frames constructed using MATLAB R2014a (http://www.mathworks.com/).

Movie S2. Sequence of satellite-derived chlorophyll concentration images with the altimetry-inferred long- (solid) and short-term (dashed) resilient material loops enclosing LCR *Kraken* overlaid. Animation constructed using FFmpeg (http://www.ffmpeg.com); individual frames constructed using MATLAB R2014a (http://www.mathworks.com/).

Movie S3. Snapshots of the evolution of satellite-tracked surface drifters (blue) along with that of the altimetry-inferred coherent Lagrangian ring long- (solid red) and short-term (dashed red) boundaries of LCR *Kraken*. The drifter positions on each day shown are indicated by blue dots. The tails attached to the dots are week-long past trajectory segments. One point on the long-term boundary is highlighted by a black dot surrounded by a circle, for reference. Animation constructed using FFmpeg (http://www.ffmpeg.com); individual frames constructed using MATLAB R2014a (http://www.mathworks.com/).
